# Arthropod-Borne Viruses in Mauritania: A Literature Review

**DOI:** 10.3390/pathogens12111370

**Published:** 2023-11-20

**Authors:** Abdallahi El Ghassem, Bedia Abdoullah, Jemila Deida, Mohamed Aly Ould Lemrabott, Mohamed Ouldabdallahi Moukah, Mohamed Salem Ould Ahmedou Salem, Sébastien Briolant, Leonardo K. Basco, Khyarhoum Ould Brahim, Ali Ould Mohamed Salem Boukhary

**Affiliations:** 1 Unité de Recherche Génomes et Milieux, Faculté des Sciences et Techniques, Université de Nouakchott, Nouakchott BP 880, Mauritania; abdellahielghassem@gmail.com (A.E.G.); bediaabdoullah@gmail.com (B.A.); jemilasidiali@gmail.com (J.D.); mohamedalylemrabott@yahoo.fr (M.A.O.L.); hmoukah2002@yahoo.fr (M.O.M.); salem0606@yahoo.fr (M.S.O.A.S.); khyarhoum.brahim@gmail.com (K.O.B.); 2 Unité de Parasitologie Entomologie, Département de Microbiologie et Maladies Infectieuses, Institut de Recherche Biomédicale des Armées (IRBA), 13005 Marseille, France; sbriolant@wanadoo.fr; 3 Institut de Recherche pour le Développement (IRD), Assistance Publique-Hôpitaux de Marseille (AP-HM), Service de Santé des Armées (SSA), Vecteurs—Infections Tropicales et Méditerranéennes (VITROME), Aix Marseille Université, 13005 Marseille, France; lkbasco@yahoo.fr; 4 Institut Hospitalo-Universitaire (IHU)-Méditerranée Infection, 13005 Marseille, France

**Keywords:** arboviruses, Crimean–Congo hemorrhagic fever, dengue, epidemics, Mauritania, mosquitoes, outbreaks, Rift Valley fever, ticks

## Abstract

During the past four decades, recurrent outbreaks of various arthropod-borne viruses have been reported in Mauritania. This review aims to consolidate the current knowledge on the epidemiology of the major arboviruses circulating in Mauritania. Online databases including PubMed and Web of Science were used to retrieve relevant published studies. The results showed that numerous arboviral outbreaks of variable magnitude occurred in almost all 13 regions of Mauritania, with Rift Valley fever (RVF), Crimean–Congo hemorrhagic fever (CCHF), and dengue (DEN) being the most common infections. Other arboviruses causing yellow fever (YF), chikungunya (CHIK), o’nyong-nyong (ONN), Semliki Forest (SF), West Nile fever (WNF), Bagaza (BAG), Wesselsbron (WSL), and Ngari (NRI) diseases have also been found circulating in humans and/or livestock in Mauritania. The average case fatality rates of CCHF and RVF were 28.7% and 21.1%, respectively. RVF outbreaks have often occurred after unusually heavy rainfalls, while CCHF epidemics have mostly been reported during the dry season. The central and southeastern regions of the country have carried the highest burden of RVF and CCHF. Sheep, cattle, and camels are the main animal reservoirs for the RVF and CCHF viruses. *Culex antennatus* and *Cx. poicilipes* mosquitoes and *Hyalomma dromedarii*, *H. rufipes*, and *Rhipicephalus everesti* ticks are the main vectors of these viruses. DEN outbreaks occurred mainly in the urban settings, including in Nouakchott, the capital city, and *Aedes aegypti* is likely the main mosquito vector. Therefore, there is a need to implement an integrated management strategy for the prevention and control of arboviral diseases based on sensitizing the high-risk occupational groups, such as slaughterhouse workers, shepherds, and butchers for zoonotic diseases, reinforcing vector surveillance and control, introducing rapid point-of-care diagnosis of arboviruses in high-risk areas, and improving the capacities to respond rapidly when the first signs of disease outbreak are identified.

## 1. Introduction

Vector-borne diseases (VBDs) account for 17% of infectious diseases globally, affecting millions of people around the world and causing 700,000 deaths annually [1]. VBDs are caused by various types of pathogenic agents, such as bacteria, parasites, and viruses, transmitted to humans or animals by different hematophagous arthropods, including mosquitoes, ticks, and other insects [2]. Among pathogenic agents, viruses transmitted by arthropods, also known as arboviruses, represent a significant burden to public health and local economies owing to their ability to cause unpredictable and widespread epidemics [3].

The major human arboviruses are single-stranded positive- or negative-sense RNA viruses belonging to four families, *Togaviridae*, *Flaviviridae*, *Phenuiviridae*, and *Nairoviridae* [4]. They are responsible for epidemics and epizootics, e.g., yellow fever (YF), Rift Valley fever (RVF), Crimean–Congo hemorrhagic fever (CCHF), dengue fever (DF), Zika (ZIK) and chikungunya (CHIK), to mention a few [5,6]. Most of the known arboviral diseases are of zoonotic origin, i.e., the natural transmission cycle is maintained by a vector and a wild animal [7]. The zoonotic transmission of these viruses occurs either through direct contact with biological materials from a viremic animal or through the bites of infected arthropods. Human-to-human transmission can also occur, mostly among medical staff in resource-poor hospitals [8].

The Islamic Republic of Mauritania is a large (1,030,700 km^2^) and sparsely populated (4 inhabitants/km^2^) West African country located between the 15th and 27th degree North latitude and the 5th and 17th degree West longitude at the interface between the Maghreb and sub-Saharan African countries. Two-thirds of the surface area of Mauritania is occupied by the Sahara Desert, and the remaining one-third is part of the Sahel. The amount of rainfall is moderate (250 mm/year on average) in the southern Sahelian regions and low (average, 50 mm/year) in the northern Saharan zone. Throughout the country, rainfall generally occurs between July and September, with a peak between mid-August and mid-September.

During the past four decades, Mauritania has experienced numerous emerging and re-emerging arboviral epidemics, mostly transmitted through the bites of infected mosquitoes and ticks [9,10,11]. As a consequence, in 2000, the Mauritanian health authorities established a National Disease Surveillance System in five geographic regions and a notification system for hemorrhagic fevers in healthcare centers [12]. However, the outbreaks of arboviral diseases that are known to exist in the country have increased in frequency and number of affected humans or animals and even spread to regions that have been spared until recently [13]. In addition, the emergence of ‘new’ arboviruses of public health concern, such as dengue virus (DENV), in urban settings like Nouakchott, the capital city, has been recently reported [14,15].

The objective of the present literature review was to consolidate the current knowledge on the epidemiology of the main arthropod-borne viral diseases endemic in Mauritania and discuss their environmental and socioeconomic drivers and main pathways that can potentially become targets for interventions.

## 2. Materials and Methods

### 2.1. Search Strategy

Relevant articles were searched in the PubMed and Web of Science electronic databases using different combinations of the following terms/keywords and Boolean operators: “Mauritania” AND “arbovirus, arboviral infections, arthropod-borne virus” OR “Zika virus, Zika, ZIKV” OR “Dengue virus, Dengue fever, DENV” OR “Yellow fever virus, Yellow fever, YFV” OR “chikungunya virus, chikungunya, CHIKV” OR “Rift valley fever virus, RVFV, Rift valley fever” OR “Crimean Congo virus, Crimean Congo hemorrhagic fever, CCHFV” OR “West Nile virus, West Nile fever, WNV”. Additional information was obtained from the patients’ laboratory record books through the National Institute for Research in Public Health (INRSP), Ministry of Health, Mauritania.

There were no time limits, and articles identified up to 31 July 2023 were included. These should have focused on the epidemiology and ecology of arboviruses, including geographical location, study subjects (humans and/or animals), number of cases or prevalence, mortality, diagnostic method used, virus species detected, serotypes, genetic diversity, risk factors of transmission, mosquito vector and their habitats, potential host or reservoir, and/or epidemics/outbreaks. Abstracts without available full text, articles written in languages other than English or French, and duplicated information were not included in the present review.

### 2.2. Analyses

Tables and graphs were used to report the findings from the analysis, including the year of arboviral outbreak and the affected regions, the number of confirmed cases and the corresponding fatality rate, the detection method employed, and arthropod vectors and animal reservoirs involved in the arboviruses’ transmission and maintenance. A geographical map showing the distribution of arboviral outbreaks in Mauritania was also drawn.

## 3. Results and Discussion

**Figure 1:**
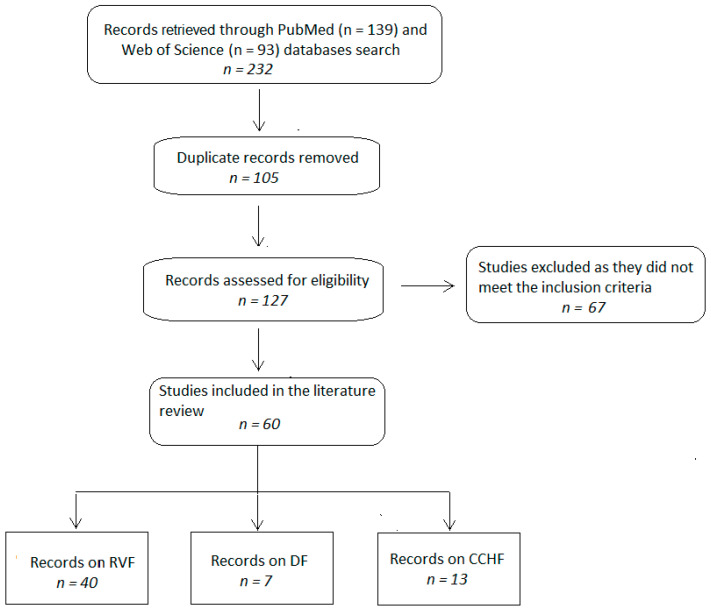
Flowchart of study selection for inclusion in the literature review.

A total of 232 published articles (93 in PubMed and 139 in Web of Science) were retrieved from the databases (Figure 1). After duplicates were removed (n = 105), the article titles and abstracts of 127 records were screened and assessed for eligibility. Following the screening process, 67 of 127 published papers were excluded as they did not meet the inclusion criteria. The remaining 60 studies were retained for the literature review covering RVF (40/60; 66.7%), CCHF (13/60; 21.7%), and DEN (7/60; 11.6%).

**Figure 2:**
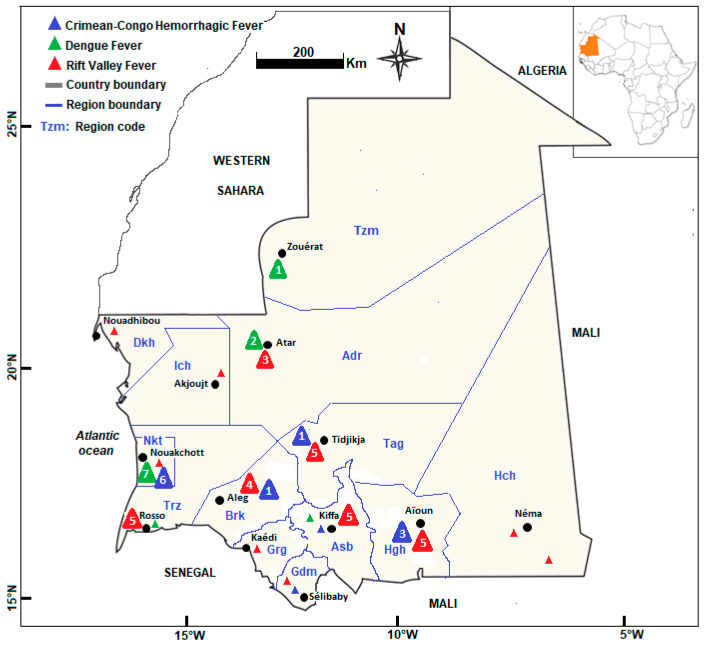
Geographical distribution of documented epidemics in Mauritania. Crimean–Congo hemorrhagic fever (blue triangles), dengue (green triangles), and Rift Valley fever (red triangles). Large triangles denote that in a given region there was at least one major outbreak in which more than 10 confirmed cases were reported, and small triangles indicate sporadic cases. The numbers inside each triangle refer to the number of reported outbreaks. Region abbreviations: Adr: Adrar; Asb: Assaba; Brk: Brakna; Dkh: Dakhlet Nouadhibou; Grg: Gorgol; Gdm: Guidimagha; Hch: Hodh Chargui; Hgh: Hodh Elgharbi; Ich: Inchiri; Nkt: Nouakchott; Tag: Tagant; Trz: Trarza; Tzm: Tiris Zemmour.

The reported cases of arthropod-borne virus infection occurred either during or immediately after acute outbreaks [10,16]. Some studies also reported sporadic cases of arthropod-borne virus infections outside the epidemic periods [17]. Various diagnostic approaches, including serology (enzyme-linked immunosorbent assay (ELISA), serum neutralization test, indirect immunofluorescent assay), rapid diagnostic tests, tissue culture (cell lines Vero or C6/36; virus inoculation in AP61 cell lines) and partial genome detection using reverse transcription polymerase chain reaction (RT-PCR), partial viral genome sequencing, and phylogenetic analysis, have been employed to diagnose and characterize arboviral infections in Mauritania [13,18,19]. Sample specimens from humans, animals, and/or mosquitoes were analyzed. Some studies reported co-occurrence or co-infections of arthropod-borne viruses, including CCHFV, RVFV, and DENV [18,19]. The geographic distribution of the RVF, CCHF, and DF outbreaks in Mauritania is shown in Figure 2.

### 3.1. Rift Valley Fever

Rift Valley fever (RVF) is a re-emerging acute viral anthropozoonosis that causes epizootics and epidemics. In addition, RVF causes heavy economic losses by affecting animal production and trade [20]. The World Organization for Animal Health (WOAH, formerly known as Office International des Epizooties (OIE)) has designated RVF as a notifiable disease [21]. The causative agent of RVF is a *Phlebovirus* belonging to the family of *Phenuiviridae*. It is transmitted to humans and domestic and wild animals by the bite of several species of mosquitoes, especially the *Aedes* and *Culex* genera, or by direct contact with the blood, body fluids, or tissues of infected animals, the latter being the main route of transmission for human infection [22]. In addition, RVFV transmission is maintained vertically among mosquito vector populations. RVFV periodically emerges to cause epizootics in livestock and epidemics in persons living nearby [23].

RVF is endemic in Mauritania, considered to be the main West African epidemic and epizootic area [16]. RVF is also present in other areas in sub-Saharan Africa as well as in the Arabian Peninsula, with repeated epidemics characterized by 5–15 years of inter-epizootic periods [24]. In Mauritania, eight major RVF outbreaks have been reported in the past four decades (1987, 1998, 2003, 2010, 2012, 2015, 2020, and 2022), representing an average of an outbreak once every 4.5 years (Table 1). They resulted in an overall case fatality rate of 21.1%. The first epidemic occurred in 1987 in Rosso, southern Mauritania, one year after the impoundment of the Diama dam on the Senegal River Valley [9,25]. During this outbreak, at least 224 humans died, and thousands of livestock, mainly small ruminants and cattle, were also lost. Serological surveys conducted a few years (1982–1985) prior to this first RVF outbreak already revealed an important circulation of the RVFV in southern Mauritania in small ruminants, cattle, camels, and humans, with 17.8% of small ruminants (83 positive sera/466 tested) presenting antibodies against the RVFV [26]. The second known RVF epidemic broke out in 1998. A significant decrease in the prevalence of anti-RVF immunoglobulin G (IgG) antibodies was reported among small ruminants in 1989 (14%), 1990 (12.4%), and 1992 (3.4%) [27].

Epidemics and epizootics of varying magnitude occurred in 1993, 1998, and 2003 involving human and animal cases [12,27,28]. The 2003 RVF outbreak covering five regions in southern, southeastern, and central Mauritania resulted in 25 confirmed human cases, including 16 with hemorrhagic signs and 4 deaths [12].

In 2010, Mauritania notified to the World Health Organization (WHO) a total of 63 human cases of RVF, including 13 deaths [13]. These cases occurred for the first time in the northern desert region of Adrar. This unexpected RVF outbreak, given its location in a hyper-arid region, occurred after atypically strong rainfalls in September 2010, which resulted in the creation of temporary water pools and large pastures, thus encouraging herders, particularly from the southern endemic regions, to drive their herds (camels and small ruminants) into these areas. According to El Mamy et al. [30], the virus was probably introduced in the affected areas via viremic animals, resulting in the establishment of a local transmission cycle amplified by mosquito populations which quickly colonized the temporary water pools. During that RVF outbreak, investigations were carried out using serum samples obtained from 83 small ruminants (70 goats and 13 sheep) and 5 sick camels from all affected localities. Immunoglobulin M (IgM) against RVFV was detected in 23 of 83 (27.7%) animals, and RVFV RNA was detected in three of five sampled camels [29]. Furthermore, RVFV was isolated from the camel sera a few months after this outbreak, highlighting the important role that camels possibly played during this outbreak [38].

The 2012 RVF outbreak resulted in 36 human cases, including 19 (52.7%) deaths and a large number of ruminants being affected in seven regions (Assaba, Brakna, Hodh Echarghi, Hodh Elgharbi, Tagant, Trarza, and Nouakchott) [18,30,31]. Entomological investigations carried out just after the outbreak resulted in the collection of various mosquito specimens belonging to thirteen species from 12 sites, including four species (*Aedes vexans*, *Culex poicilipes*, *Cx. antennatus*, and *Mansonia uniformis*) known to be RVF vectors in the sub-region. However, RVFV was not isolated from any of these mosquitoes during these investigations [18]. Serological analysis of samples collected from 159 camels and 118 cows at a slaughterhouse in Nouakchott in March 2013 using a commercial ELISA kit for RVFV showed a positive titer in 45% of camels and 16% of cows [39]. This survey provided further evidence on the susceptibility of camels to RVFV, suggesting its possible use as a sentinel animal.

In 2015, an RVF outbreak of large geographical distribution occurred in Mauritania [19,32]. During this outbreak, 57 positive cases in humans, including 12 (21.0%) deaths, were reported, but entomological investigations and serological studies on RVFV were not performed in humans and animals. In 2020, during the coronavirus disease 2019 (COVID-19) pandemic, an RVF outbreak resulted in 78 confirmed cases and 25 (33.3%) deaths [33,34]. During this outbreak, RVF in animals was also reported in the regions of Assaba, Tagant, Brakna, Trarza, Hodh Elgharbi, and Hodh Echarghi, where 74 camels, 52 small ruminants, and 12 cows were positive [35].

The latest RVF outbreak occurred during the rainy season in 2022 during which 47 confirmed cases and 23 (49.0%) deaths, mostly among animal breeders, were notified [36,37]. This outbreak was one of the deadliest documented RVF outbreaks in Mauritania. RVF-confirmed human cases occurred in 9 of 15 Mauritanian provinces. Serological studies in sentinel animals (cattle, camels, and small ruminants) across twelve provinces, including nine that share borders with Senegal, Mali, and Algeria, showed that 5.2% (5/96) of cattle and 25.9% (159/614) of small ruminants were positive according to ELISA IgM while 25.8% (113/438) of camels were positive according to RT-PCR [36].

RVFV circulation during inter-epidemic periods was also assessed in productive livestock from different regions in Mauritania. A study conducted in 2012 and 2013 covering two inter-epidemic periods showed significant serological differences between tested animals with seroprevalences of 3.8% in small ruminants (9.5% of sheep vs. 1.4% of goats when compared with each other), 15.4% in cattle, and 32% in camels [17]. RVF viral strains in Mauritania have been genotyped and have been shown to belong to both the Northeastern African (also known as Egyptian) group and East–Central African clusters found in East Africa [12].

### 3.2. Crimean–Congo Hemorrhagic Fever

CCHF is the most widespread tick-borne viral disease caused by an *Orthonairovirus* belonging to the *Nairoviridae* family. The disease was first reported from Crimea (a peninsula located on the northern coast of the Black Sea in Eastern Europe) in 1944 and later recognized as the same illness as the one reported in the Republic of the Congo (in Central Africa) in 1956, thus resulting in the current name of the disease [40,41,42].

Ixodid (hard) ticks, particularly those of the genus *Hyalomma*, are both the vector and the reservoir of the CCHFV. Numerous wild and domestic animals, such as camels, cows, goats, sheep, and hares, serve as the amplifying hosts of the virus. Transmission to humans occurs as a result of bites by infected adult ticks or through direct contact with blood, secretions, or infected tissues of viremic patients (human-to-human transmission) or livestock (animal-to-human transmission). Human-to-human transmission of CCHFV usually occurs in healthcare settings and mostly affects healthcare workers [10,43]. CCHFV causes severe hemorrhagic outbreaks with a case fatality rate of up to 40% [42]. The virus is found in Eastern Europe, particularly in the former Soviet Union, northwestern China, central Asia, throughout the Mediterranean basin, Africa, the Middle East, and the Indian subcontinent [44].

In West Africa, CCHF is endemic in southern and central Mauritania, where 10 outbreaks have been documented since the 1980s (Table 2). CCHF is also endemic in northern Senegal, the neighboring country of Mauritania [45,46]. The first human case due to CCHFV in West Africa was identified and serologically confirmed in 1983 in a camel breeder in Sélibaby, Guidimakha region, southernmost region in Mauritania, probably after close contact with infected camels or cattle [47,48]. In 1985, a serological survey conducted in different ethnic groups of Mauritania showed a very low (0.2%) prevalence of antibodies directed against CCHFV [49].

Twenty years after the first confirmed CCHF cases in Sélibaby, a second CCHF outbreak, which was the first urban CCHF outbreak in Mauritania, occurred in Nouakchott, the capital city [10]. It resulted in 38 confirmed cases, of whom 35 were residents in Nouakchott, and six deaths. This was also the first outbreak in which a nosocomial transmission of the CCHFV occurred, involving 15 members of the hospital staff and patients in the emergency ward of the same hospital.

A seroprevalence study conducted in 2013 using 495 cattle sera collected from different provinces in Mauritania showed an overall prevalence of CCHFV-IgG-specific antibodies of 67%, with a range of 56–90% according to their geographic origin [58]. More recently, a study conducted in 928 livestock animal samples (cattle, n = 201; sheep, n = 247; goats, n = 233; camels, n = 247) covering 11 of 13 provinces of Mauritania using CCHFV-specific IgG antibodies showed that 15% of small ruminants (goats and sheep) were seropositive, whereas in cattle (69%) and camels (81%), the prevalence rate was significantly higher [59]. More interestingly, this study showed that the seroprevalence in all species was age-dependent, i.e., older animals had significantly higher seroprevalence rates than younger animals.

Between 4 February and 14 March 2022, seven CCHF cases, including two (28.5%) deaths, were reported in Mauritania, including one animal breeder from the department of Kobeni in the south-eastern region of Hodh El Gharbi [56,57]. During this outbreak, *Hyalomma* ticks were found on cattle, sheep, and goats in the areas where the patients resided, and one of the infected patients was in close contact with fresh animal meat [56]. Viral RNA sequence analysis of the S segment of all positive samples revealed the presence of two different CCHFV lineages, Africa I (Senegal) and Africa III (Mauritania/Mali) [59]. The presence of multiple lineages of CCHFV in Mauritania could be the result of the annual livestock (small and large ruminants) transhumance in vast areas of the Sahel, including Mali, Senegal, and Mauritania, during the long dry season in which viremic animals can disseminate the virus in large areas of pasture. It also suggests that CCHFV strains are not strictly restricted to a defined geographic location, but considerable mobility of viruses can occur over long distances through bird migration [60].

### 3.3. Dengue Fever

DF is the most widespread tropical and subtropical mosquito-borne viral disease causing more than 96 million symptomatic cases and approximately 40,000 deaths per year [61]. DF is an arboviral disease caused by the DENV, an arbovirus belonging to the family *Flaviviridae*, genus *Orthoflavivirus*. *Aedes aegypti* mosquito is the primary vector of urban DENV. *Aedes albopictus* also contributes to the transmission of DENV as a secondary vector [62]. These mosquito vectors have become widely distributed across tropical and subtropical regions and spread globally with the advent of various phenomena, including urbanization, rapid demographic growth, inadequate water supply, poor sewerage system, poor sanitation, and international travel. DF is asymptomatic in more than 50% of cases or presents as a flu-like illness, including headache, myalgia, and rash, as in other febrile diseases in Africa like malaria and chikungunya fever [63].

The first laboratory-confirmed dengue outbreak in Mauritania occurred during October–November 2014 in Nouakchott, where an upsurge in the number of febrile patients with flu-like symptoms suggestive of malaria was reported in health centers and hospitals [14,15]. During that period, which coincided with the peak of the malaria transmission season, all tested patients had a negative rapid diagnostic test for malaria. The results of the rapid diagnostic test for dengue fever (SD Bioline Dengue non-structural protein 1 (NS1) + IgG/IgM) performed in some of these febrile patients were positive, and serological tests conducted in the Pasteur Institute in Dakar (Senegal) confirmed the presence of DENV (El Bara, personal communication). In addition, a recent study confirmed the presence of DENV in two DF cases according to quantitative RT-PCR using blood samples collected during the outbreak in 2014 in Mauritania [64]. A few months before the first DF outbreak in the capital city, *Ae. aegypti* mosquitoes were found for the first time in Nouakchott [65].

The emergence of DF during the same period, i.e., around 2014, is also supported by the passive surveillance system of imported dengue in France carried out by the French National Reference Center for Arboviruses (Marseille, France) that reported one PCR-confirmed DENV serotype 1 (DENV-1) case in a French traveler returning from Mauritania in October 2015 [66].

Since its first detection in 2014, DF outbreaks with different magnitudes and limited geographical distribution have occurred annually in Mauritania (Table 3). Patient records from these outbreaks are filed and stored in a database held by the INRSP in Nouakchott, Mauritania, and some of these data have been published by the WHO [67]. Briefly, 302, 291, 79, 32, 307, 14, 8, and 11 confirmed cases were reported in 2014, 2015, 2016, 2017, 2018, 2019, 2020, and 2022, respectively (INRSP, unpublished data). However, due to the fact that a vast majority of dengue cases are mild and self-limited and most health centers do not offer dengue diagnostic tests, the actual number of dengue cases is most likely under-reported. Furthermore, in sub-Saharan African countries, many mosquito-transmitted arboviruses, such as dengue and chikungunya, remain undiagnosed or are often misdiagnosed as malaria [68].

Between 2014 and 2017, DF cases were reported only in Nouakchott. From 2018, DF spread rapidly throughout the country, particularly to the northern Saharan cities, where it was detected in Zouérat, a northern mining city, and in the oasian city of Atar, where entomological investigations showed the presence of *Ae. aegypti* mosquitoes during these outbreaks (Ould Ahmedou Salem, personal communication).

The 2018 outbreak was the most widespread among all DF epidemics that occurred in Mauritania, which explains the high number of DF cases (307 cases confirmed by the rapid diagnostic test for dengue) recorded during that outbreak. Indeed, of 65 PCR-confirmed DF cases collected from four regions in Mauritania, the majority (47/65; 72%) were from Nouakchott, 9 (14%) from Rosso district in the Trarza region (southern Mauritania), 7 (11%) from Guérou district in the southern Assaba region, and 2 (3%) from Zouérat district in the Tiris Zemmour Region (northern Mauritania). Among these confirmed cases, dengue hemorrhagic fever or fatalities associated with DF have not been registered [67]. Because of the absence of data on the travel history of the DF-confirmed patients during this outbreak, the origin of DF cases diagnosed outside Nouakchott cannot be determined.

DENV-1 and DENV serotypes 2 (DENV-2) were reported in Mauritania [15,64,69]. The country was free of dengue at least until 2014. It is still not clear how DENV and its anthropophilic vector *Ae. aegypti* were introduced into Mauritania. However, phylogenetic analysis of Mauritanian isolates of DENV suggested a recent emergence of epidemic strains belonging to group V Asian lineage for the DENV-1 serotype [66] and West African cosmopolitan lineage II for DENV-2 [15]. Further collection of DENV strains and sequencing analysis are required to understand how Mauritanian strains of DENV are related to other DENV strains circulating in West Africa and elsewhere in the world.

### 3.4. Other Arboviruses

Other arboviruses affecting humans as well as livestock have occasionally been reported in Mauritania (Table 4). Among the more well-known arboviruses are the YF and WNF viruses. During the first RVF epidemic in Mauritania in 1987, 24 YF cases were diagnosed and confirmed (23 by the detection of specific IgM and 1 by virus isolation) [70]. In addition, a sero-surveillance study also reported the circulation of WNV in camels with a prevalence of 92%, suggesting the important role that camels play as an animal reservoir of this virus in the country [39].

Other less known arboviruses reported in Mauritania include Bagaza virus (flavivirus) isolated from *Cx. neavei* and *Cx. poicilipes*, Wesselsbron virus (flavivirus) isolated from *Ae. vexans* [16,19], and Ngari virus (orthobunyavirus) isolated from goats and sheep during the 2010 and 2015–2016 RVF outbreaks in the Adrar region, northern Mauritania [71,72]. Other serological studies have shown the presence of two viruses belonging to the genus *Orbivirus*, epizootic hemorrhagic disease virus, an epizootic arboviral disease of wild ungulates [73], and African horse sickness virus, which can cause a life-threatening hemorrhagic disease in equids [74], both transmitted between hosts primarily by biting midges of the genus *Culicoides* (Diptera: *Ceratopogonidae*). These Orbiviruses have been demonstrated with 73% and 3% seroprevalence, respectively [39].

More recently, a cross-sectional seroprevalence study involving 1300 non-febrile patients consulting at the Nouakchott hospital center during 2021 reported for the first time serological evidence of previous exposure to chikungunya virus (CHIKV) with a seroprevalence of 2.8% and co-circulation of two other alphaviruses, o’nyong-nyong virus (ONNV) and Semliki Forest virus (SVF), in Nouakchott with a seroprevalence of 2.3% in the study population [75].

**Table 4:** Reported arboviruses circulating in humans, animals, and arthropods in Mauritania.

Human/Animal/Arthropod		AHSV	BATV	BUNV	BAGV	CCHFV	CHIKV	DENV	EHV	NRIV	ONNV	RVFV	SFV	WNV	WSL	YFV	Reference
Humans						+	+	+			+	+	+	+		+	[15,19,54,70,75]
Animals																	
	Cattle					+						+					[34,39,59]
	Dromedary	+				+			+			+		+			[29,39,59]
	Goats		+			+				+		+					[10,71,72]
	Sheep		+	+		+				+		+					[10,72]
Arthropods																	
	*Aedes vexans*											+			+		[16,19]
	*Aedes aegypti*							+									[69]
	*Anopheles pharoensis*											+					[69]
	*Culex antennatus*											+					[30]
	*Culex poicilipes*				+							+					[12,16]
	*Culex neavei*				+												[16]
	*Culex quinquefasciatus*							+									[69]
	*Hyalomma dromedarii*					+											[59]
	*Hyalomma marginatum*					+											[47]
	*Hyalomma rufipes*					+											[59]
	*Hyalomma truncatum*					+											[1]
	*Rhipicephalus eversti eversti*					+											[10]

Key: + screened positive for virus or virus antibodies; AHSV: African horse sickness virus; BATV: Batai virus; BUNV: Bunyamwera virus; BAGV: Bagaza virus; CCHFV: Crimean–Congo hemorrhagic fever virus; CHIKV: chikungunya virus; DENV: dengue fever virus; EHV: Epizootic Hemorrhagic virus; NRIV: Ngari virus; ONNV: o’nyong-nyong virus; RVFV: Rift Valley fever virus; SFV: Semliki Forest virus; WNV: West Nile virus; WSLV: Wesselsbron virus; YFV: yellow fever virus.

### 3.5. Arthropod Vectors and Animal Reservoirs of Arboviruses in Mauritania

Various arthropod vectors and animal reservoirs are involved in the transmission and/or maintenance of arboviruses in Mauritania (Table 4). *Culex poicilipes*, *Ae. vexans*, *Anopheles pharoensis*, and *Cx. antennatus* are potential mosquito vectors of RVFV in Mauritania, as specimens of these mosquito species were found naturally infected with the virus during the 1998–1999, 2003, and 2010 RVF outbreaks [12,16,29,69]. Ticks belonging to the genus *Hyalomma* are the main arthropod vectors of the CCHFV circulating in Mauritania. Indeed, one year after the first human CCHF case in 1983, epidemiological surveys conducted in Sélibaby and its surrounding areas resulted in the isolation of five CCHFV strains in ticks (*Hyalomma marginatum rufipes*) collected from camels [59].

Using a one-step multiplex real-time RT-PCR, Schulz et al. [59] found that 2.6% of *H. dromedarii* and *H. rufipes* ticks collected from camels and cattle were positive for CCHFV. No *H. impeltatum* ticks tested positive during this survey.

The simultaneous presence of DENV and the primary vector known to transmit DENV to humans worldwide, *Ae. aegypti*, implies that, although there is at present no solid entomological evidence that this mosquito species is the principal vector of dengue in Nouakchott since the virus has never been isolated from the mosquito in Mauritania, it can safely be assumed that *Ae. aegypti* is involved in DENV transmission in the country. In a recent study, the evidence of DENV circulation and transmission was found in a pool of eight female *Ae. aegypti* mosquitoes collected in December 2018 in the city of Rosso, south-western Mauritania, as well as in a pool of ten female *Cx. quinquefasciatus* mosquitoes [69]. The latter finding in *Culex* is probably artefactual, but further confirmatory entomological studies are needed to ensure that other mosquito species are not involved in DENV transmission.

West Nile virus (WNV) was isolated from *Ae. vexans* [16,19]. Bagaza virus was isolated from *Cx. neavei* and *Cx. poicilipes*, Wesselsbron virus was isolated from *Ae. vexans* [16,19], and Ngari virus was isolated from goats and sheep during the 2010 and 2015–2016 RVFV outbreaks in the Adrar region, northern Mauritania [71,72]. The impact of these arboviruses on human and animal health is yet to be evaluated.

### 3.6. Factors Associated with Arbovirus Endemicity in Mauritania

#### 3.6.1. Climatic and Environmental Conditions

Historically, the emergence of RVF outbreaks in Mauritania has been closely associated with climatic conditions, particularly unusually strong and/or delayed rainfalls leading to flooding and hatching of large numbers of mosquito larvae and subsequent emergence of adult mosquitoes that feed on viremic animals [30,76,77]. For instance, during the first RVF outbreak that occurred in Mauritania and probably in West Africa in 1987, rainfall in Rosso and the surrounding areas where the RVF outbreak occurred was abundant, heterogeneously distributed, and unseasonably late [78,79]. In addition, flooding of the Senegal River basin following the completion of the Diama hydro-agricultural dam increased the number of potential mosquito breeding sites in areas where the virus was already known to be present. During severe drought conditions, it also led to the concentration of people and livestock in the proximity of the dams [80].

Similar climatic and environmental conditions were observed during subsequent RVF outbreaks, particularly those that occurred in the hyper-arid northern zone of Mauritania in 2010 where exceptionally heavy rainfall was noted in many affected localities [13] and in southern Mauritania where a two-fold increase in the amount of rainfall was recorded in 2012 compared to 2011 [18].

Studies on RVF outbreaks in West Africa showed that they usually occur during the late rainy season [81]. A strong association between the occurrence of unusually prolonged and heavy rainfalls and the emergence of RVF outbreaks was also shown [81]. In Mauritania, like in Senegal, RVF outbreaks generally occur during the months of September, October, and November, corresponding to the end of the rainy season and the beginning of the dry season [19,34,77]. Most RVF epidemics were preceded by a rainless period of at least one week followed by heavy rainfall [13,28,29].

Unlike RVF, where outbreaks commonly occur during or shortly after the rainy season, CCHF outbreaks reported in humans in Mauritania mostly occurred during the long dry season (i.e., December–July) [56]. Globally, CCHF outbreaks have been reported in areas characterized by year-long warm temperatures, low precipitation, and moderate annual humidity as those prevailing in the Sahelian region in Africa [44,82] where *Hyalomma marginatum* ticks abound [83]. Furthermore, evidence from modelling studies conducted in Europe to explain and predict *H. marginatum* tick distribution suggested that its most suitable habitats are characterized by high temperatures, low precipitation, and low relative humidity [82,84,85]. In Mauritania, as well as in other Sahelian countries, these conditions prevail during the hot and dry seasons (April–June) during which most CCHF outbreaks have been reported. Furthermore, available evidence suggests that *H. impeltatum*, another potential vector of CCHFV, is most abundant in arid areas, and its numbers decrease with increasing rainfall [86].

An upsurge in the number of DF cases is usually reported during or at the end of the rainy season (September–November). This observation is not necessarily associated with rainfall. An increased availability and creation of mosquito larval habitats in an urban setting (e.g., abandoned used tires, water pools around public standpipes, wells, water storage containers without covers) favor the development of the larvae of anthropophilic dengue vector *Ae. aegypti* [65,87]. More interestingly, DF outbreaks have occurred so far only in the urban areas of the country (i.e., Nouakchott, Atar, and Zouérat) where artificial containers abound around the houses for individual water supply reservoirs and serve as mosquito larval habitats for *Ae. aegypti* larvae [58]. Similar findings were reported from urban sites in Côte-d’Ivoire [88] and everywhere else where DENV is endemic.

#### 3.6.2. Role of Livestock

Mauritania has a livestock population of 19 million small ruminants (sheep and goats), 1.9 million cattle, and 1.5 million camels [89,90]. Bovine livestock and the majority of the herds of small ruminants are raised in the southern Sahelian zone. By contrast, herds of camels and a minority of small ruminants (mainly goats) are traditionally grazed for the most part on pastures in the saharo-sahelian zones and along the Atlantic coast and are conducted to different areas across the Sahara Desert by transhumance [91]. Nomadic and transhumant pastoralism is the most dominant livestock farming system in Mauritania. Transhumance is regularly practiced not only within Mauritania but also between Mauritania and its neighboring countries (i.e., Senegal and Mali), particularly during the last months of the long dry season (April–June), either to lead livestock to better grazing areas [92] or to sell their meat at livestock markets [93,94]. Furthermore, the high demand for camel milk and meat created important commercially oriented peri-urban camel farming around urban settings, particularly in Nouakchott, potentially increasing the risk of camel-to-human transmission of viruses through mosquito vectors or by direct transmission.

Several studies have demonstrated a direct relationship between the traditional livestock farming system involving sheep, cattle, and camels, which requires close contact between animal breeders and these animals acting as reservoirs for the virus, and the transmission of some arboviral diseases [95]. Among domestic animals present in Mauritania, the susceptibility of sheep, cattle, and camels to RVFV and CCHFV has been reported (Table 4). Based on these observations, it has been established that these animals can be infected with these viruses, develop viremia, and amplify the viruses, thus playing an important role in the maintenance of CCHFV and RVFV circulation [96].

Direct contact with blood, body fluids, or tissues of infected animals is the dominant risk factor for animal-to-human transmission of CCHFV and RVFV in Mauritania [10]. In fact, most of the documented RVF and CCHF epidemics in Mauritania were caused by close contact with blood or fresh organs of infected animals during slaughtering or when assisting female animals during delivery. This is supported by the fact that at least half of the index patients during the CCHF and RVF outbreaks in Mauritania were butchers, and many other index patients were housewives, suggesting that handling freshly cut meat is a risk for infection [10,13,54,56].

In addition, the long-distance migration of domestic animals during the long dry season could be associated with the diffusion of RVFV and CCHFV through livestock by introducing viremic animals to virus-free areas for possible animal-to-human transmission by direct contact or by infecting arthropods [56]. Hence, as more and more livestock become infected with arboviruses, the risk of transmission to humans increases. In addition, during the wet season, corresponding to the summer vacation period, Mauritanians tend to spend more time in rural areas where RVF transmission occurs more often than in urban areas, often resting in the open air until late at night, resulting in an additional risk factor for exposure to mosquitoes.

#### 3.6.3. Rapid and Disorganized Urbanization

Poorly planned development, rapid urbanization, and increasingly dense human populations, in combination with climate and environmental changes, provide favorable conditions for the creation of habitats for some species of mosquito and increase the risk of transmission of certain diseases [97]. Besides the high density of people that characterizes unplanned urban settings, the presence of domestic and peri-domestic animals further contributes to the spread of arboviral diseases of zoonotic origin [98].

The current demography and urban organization of Nouakchott is the result of several decades of uncontrolled and unplanned urbanization following the major long-lasting drought in the Sahel during the 1970s and 1980s, resulting in a massive rural exodus. Created de novo in the late 1950s as the capital of Mauritania, with only 500 inhabitants initially, Nouakchott is currently one of the most densely populated cities in the Sahara with 1000 inhabitants/km^2^ [99,100]. The scarcity and quality of safe potable water supply were among the numerous challenges that the city planners of Nouakchott had to face over the past four decades. To palliate this problem, numerous water points were created to supply potable water to newly settled populations residing in the slums surrounding the city center. However, inadequate management of these water sources led to the creation of countless numbers of puddles around them, resulting in the creation and establishment of mosquito habitats, particularly *An. gambiae* sensu lato (s.l.) and *Ae. aegypti* [65,101]. Both of these mosquito species spread rapidly in the capital city, leading to not only regular dengue epidemics but also malaria epidemics [102,103]. The problem associated with water leaks from pipes and puddles around water points is compounded by the absence of a water evacuation system in the city, leading to large-scale flooding that may last for months after an exceptionally heavy rainfall [104].

## 4. Conclusions

The present literature review indicates that CCHFV, RVFV, and DENV are widely circulating in Mauritania. The presence of other arboviruses, including CHIKV, ONNV, YFV, and WNV, has also been reported in the literature, but recent epidemiological data on these viruses are limited. The continual resurgence of arboviruses suggests that these viruses will continue to produce unpredictable local and even regional outbreaks and pose the problem of the effectiveness and sustainability of the current control strategies in Mauritania, as well as in the West African sub-region. Therefore, there is an urgent need to implement an integrated management strategy for the prevention and control of arboviral diseases. Among the priority measures, mass sensitization campaigns targeting the high-risk groups, such as slaughterhouse workers, shepherds, and butchers, and an early warning system for reporting suspicious animal health problems are particularly important measures that should be developed to contain zoonotic diseases before they are transmitted to humans and spread among people. Furthermore, vector control should be reinforced. Since *Ae. aegypti* mosquitoes preferentially develop larval habitats around the home in artificial containers or in used tires abandoned in dumpsites, vector control efforts should focus on the reduction in larval sources through sustained community participation. Furthermore, the co-circulation of other arboviruses in the country calls for a surveillance system for hemorrhagic fever and the introduction of systematic diagnostic testing for febrile illnesses to prevent a large-scale outbreak.

## Figures and Tables

**Table 1:** Rift Valley fever outbreaks in humans, Mauritania (1987–2022).

Year	Region ^a^	No. Confirmed Cases	No. Deaths	Fatality Rate (%)	Detection Method	Reference
1987	Trz	1200 ^b^	224	18.6	IgM detection, virus isolation	[9,25]
1998	Hgh	15	2	13.3	IgM and IgG detection, virus isolation, RT-PCR	[28]
2003	Asb, Brk, Tag, Trz	25 ^c^	4	16.0	IgM and IgG detection, virus isolation, RT-PCR, genome partial sequencing	[12]
2010	Ich, Adr	63	13	20.6	IgM and IgG detection, RT-PCR, virus isolation	[13,29]
2012	Asb, Brk, Grg, Hch, Hgh, Tag, Trz	36	19	52.7	IgM and IgG detection, RT-qPCR	[18,30,31]
2015	Asb, Brk, Dkh, Grg, Hch, Hgh, Nkt, Tag, Trz	57	12	21.0	IgM detection, RT-qPCR, viral genome partial sequencing	[19,32]
2020	Adr, Asb, Brk, Gdm, Grg, Hgh, Hch, Nkt, Tag, Trz	78	25	32.0	IgM detection, RT-qPCR, viral genome partial sequencing	[33,34,35]
2022	Adr, Asb, Dkh, Hch, Hgh, Nkt, Tag	47	23	49.0	IgM detection, RT-qPCR	[36,37]
1987–2022		1519	320	21.1		

Abbreviations: ELISA: enzyme-linked immunosorbent assay; IgG: immunoglobulin G; IgM: immunoglobulin M; IIFT: indirect immunofluorescence test; INRSP-PRB: National Institute of Research in Public Health patient record books; RT-qPCR: reverse transcriptase quantitative polymerase chain reaction; RDT: rapid diagnostic test; RT-PCR: reverse transcriptase polymerase chain reaction. ^a^ Region abbreviations: Adr: Adrar; Asb: Assaba; Brk: Brakna; Dkh: Dakhlet Nouadhibou; Grg: Gorgol; Gdm: Guidimagha; Hch: Hodh Chargui; Hgh: Hodh Elgharbi; Ich: Inchiri; Nkt: Nouakchott; Tag: Tagant; Trz: Trarza; Tzm: Tiris Zemmour. ^b^ During this outbreak, 24 yellow fever cases were also confirmed (23 by IgM detection and 1 by virus isolation). ^c^ Including 16 cases with hemorrhagic forms.

**Table 2:** Crimean–Congo hemorrhagic fever outbreaks in humans, Mauritania (1983–2022).

Year	Region ^a^	No. Confirmed Cases	No. Deaths	Fatality Rate (%)	Detection Method	Reference/Source
1983	Gdm	1	0	0	Serology	[47]
1988	Trz	6	1	16.7	IgM and IgG detection, virus isolation	[50]
2003	Brk, Hgh, Nkt	38	11	28.6	ELISA, RT-PCR, virus isolation/genome partial sequencing	[10]
2012	Nkt	3	0	0	ELISA/RT-qPCR	INRSP-PRB
2015	NG ^b^	6	2	33.3	IgM detection, RT-PCR, genome partial sequencing	[19,51], INRSP-PRB
2017	Nkt, Trz	4	0	0	ELISA, RT-qPCR	[52,53]
2018	Tag, Nkt, Hch	4	2	50.0	RT-qPCR	INRSP-PRB
2019	Asb, Gdm, Hgh, Nkt	6	1	16.6	IgM detection, RT-qPCR	[54,55], INRSP-PRB
2020	Hch, Trz	4	4	100.0	IgM detection, RT-qPCR	INRSP-PRB
2022	Hgh, Nkt, Trz	8	2	25.0	ELISA/RT-qPCR	[56,57]
1983–2022		80	23	28.7		

Abbreviations: ELISA: enzyme-linked immunosorbent assay; IgG: immunoglobulin G; IgM: immunoglobulin M; INRSP-PRB: National Institute of Research in Public Health patient record books; RT-qPCR: reverse transcriptase quantitative polymerase chain reaction; RDT: rapid diagnostic test; RT-PCR: reverse transcriptase polymerase chain reaction. ^a^ Region abbreviations: Adr: Adrar; Asb: Assaba; Brk: Brakna; Dkh: Dakhlet Nouadhibou; Grg: Gorgol; Gdm: Guidimagha; Hch: Hodh Chargui; Hgh: Hodh Elgharbi; Ich: Inchiri; Nkt: Nouakchott; Tag: Tagant; Trz: Trarza; Tzm: Tiris Zemmour. ^b^ Including two confirmed cases of CCHF diagnosed in the Dakar Le Dantec Hospital (Senegal) in Mauritanian patients who came from Nouakchott.

**Table 3:** Dengue fever outbreaks in humans, Mauritania (2014–2022).

Year	Region ^a^	No. Confirmed Cases	No. Deaths	Fatality Rate (%)	Detection Method	Reference/Source
2014	Nkt	302	0	0	RDT ^d^	[67]
2015	Nkt	291	0	0	RDT	INRSP-PRB
2016	Nkt	79	0	0	RDT	INRSP-PRB
2017	Nkt	32 ^b^	0	0	RDT, ELISA, RT-qPCR	INRSP-PRB
2018	Asb, Dkh, Nkt, Trz	119	0	0	RDT, RT-qPCR	[60], INRSP-PRB
2019	Nkt, Trz	14	0	0	RDT, RT-qPCR	INRSP-PRB
2020	Adr, Nkt, Tzm	8	0	0	RT-qPCR	INRSP-PRB
2022	Adr	11 ^c^	0	0	IgM detection	INRSP-PRB
2014–2022		856	0	0		

Abbreviations: ELISA: enzyme-linked immunosorbent assay; INRSP-PRB: National Institute of Research in Public Health patient record books; RT-qPCR: reverse transcriptase quantitative polymerase chain reaction; RDT: rapid diagnostic test; ^a^ Region abbreviations: Adr: Adrar; Asb: Assaba; Brk: Brakna; Dkh: Dakhlet Nouadhibou; Grg: Gorgol; Gdm: Guidimagha; Hch: Hodh Chargui; Hgh: Hodh Elgharbi; Ich: Inchiri; Nkt: Nouakchott; Tag: Tagant; Trz: Trarza; Tzm: Tiris Zemmour. ^b^ Including one case with dengue hemorrhagic fever confirmed in a Mauritanian native returning from Angola. ^c^ Of 13 suspected cases in Atar, 11 were positive using serology. ^d^ The rapid diagnostic test used was Dengue Combo NS1 and IgM/IgG.
